# Optimising quantitative ^90^Y PET imaging: an investigation into the effects of scan length and Bayesian penalised likelihood reconstruction

**DOI:** 10.1186/s13550-019-0512-y

**Published:** 2019-05-10

**Authors:** Nathaniel P. Scott, Daniel R. McGowan

**Affiliations:** 1 Radiation Physics and Protection, Churchill Hospital, Oxford University Hospitals NHS Foundation Trust, Old Road, Oxford, OX37LE UK; 2 Department of Oncology, University of Oxford, Old Road Campus Research Building, Oxford, UK

**Keywords:** Quantitative PET, Image reconstruction, Yttrium-90 PET, Bayesian penalised likelihood reconstruction, PET acquisition length

## Abstract

**Background:**

Positron emission tomography (PET) imaging of ^90^Y following selective internal radiation therapy (SIRT) is possible, but image quality is poor, and therefore, accurate quantification and dosimetry are challenging. This study aimed to quantitatively optimise ^90^Y PET imaging using a new Bayesian penalised likelihood (BPL) reconstruction algorithm (Q.Clear, GE Healthcare). The length of time per bed was also investigated to study its impact on quantification accuracy.

**Methods:**

A NEMA IQ phantom with an 8:1 sphere-to-background ratio was scanned overnight on a GE Discovery 710 PET/CT scanner. Datasets were rebinned into varying lengths of time (5–60 min); the 15-min rebins were reconstructed using BPL reconstruction with a range of noise penalisation weighting factors (beta values). The metrics of contrast recovery (CR), background variability (BV), and recovered activity percentage (RAP) were calculated in order to identify the optimum beta value. Reconstructions were then carried out on the rest of the timing datasets using the optimised beta value; the same metrics were used to assess the quantification accuracy of the reconstructed images.

**Results:**

A beta value of 1000 produced the highest CR and RAP (76% and 73%, 37 mm sphere) without overly accentuating the noise (BV) in the image. There was no statistically significant increase (*p* < 0.05) in either the CR or RAP for scan times of > 15 min. For the 5-min acquisitions, there was a statistically significant decrease in RAP (28 mm sphere, *p* < 0.01) when compared to the 15-min acquisition.

**Conclusion:**

Our results indicate that an acquisition length of 15 min and beta value of 1000 (when using Q.Clear reconstruction) are optimum for quantitative ^90^Y PET imaging. Increasing the acquisition time to more than 15 min reduces the image noise but has no significant impact on image quantification.

**Electronic supplementary material:**

The online version of this article (10.1186/s13550-019-0512-y) contains supplementary material, which is available to authorized users.

## Background

Selective internal radiation therapy (SIRT) involves the administration of ^90^Y microspheres, via the hepatic artery, for the treatment of irresectable liver malignancies [1]. Prior to treatment, the administration and imaging of ^99m^Tc-MAA (microaggregated albumin) particles, an analogue of ^90^Y microspheres, is carried out. This is to ensure that no extrahepatic migration of the microspheres occurs, and it allows an assessment of microspheres shunting to the lungs to be made [2]; extrahepatic deposition or a high lung shunt can be contraindications for treatment [3–6]. It is typically assumed that the distribution of the ^99m^Tc-MAA will match the final ^90^Y microsphere distribution and can therefore also be used as a basis for dosimetry calculations [7–9]. However, several studies have shown that this is not always the case due to differences in particle characteristics and the variation in where the microspheres are introduced [10, 11]. Therefore, the validation of the ^90^Y microsphere distribution post-SIRT is key in order to perform accurate measures of absorbed dose.

Validation can be carried out via hybrid PET/CT imaging which utilises the positron branching ratio of the ^90^Y decay chain [12]. This type of imaging however is limited by the low positron branching ratio and the random coincidence detection of bremsstrahlung photons produced from the beta emissions; the resulting images are noisy and of a low number of counts [13, 14]. OSEM is the most commonly used reconstruction algorithm in PET [15]; this is an iterative reconstruction algorithm that breaks the raw image data up into subsets in order to speed up the reconstruction process. A limitation of this type of reconstruction is that as the number of iterations increases, the noise in the image also increases [16]; the algorithm is therefore stopped after just a few iterations. This prevents noise amplification but reduces the quantitative accuracy of the image. As such, it is not an ideal reconstruction method for quantitative ^90^Y imaging [15].

Bayesian penalised likelihood (BPL) reconstruction algorithms have been applied in ^18^F-FDG PET imaging and have been shown to improve image quality and lesion detectability [17–20]. These algorithms aim to achieve effective convergence without overly accentuating the effect that noise has on the images; this is done through noise suppression within the image reconstruction algorithm. The application of these reconstructions to imaging ^90^Y is of particular interest due to the potential to reduce the noise within these low count acquisitions. GE Healthcare’s commercial software Q.Clear is a BPL iterative reconstruction algorithm that incorporates both point spread function modelling (to improve spatial resolution) and a penalising term to control the image noise [17]. The weighting of this penalising term, beta, is the only variable that is user controllable within the reconstruction. Modified block sequential regularised expectation maximisation (BSREM) is used as the optimiser within this reconstruction; this, alongside the penalty term, allows the reconstruction to be run to effective convergence without the issue of excessive noise. The beta value has been previously optimised for visualisation of ^90^Y imaging [21], but its impact on quantification has not yet been fully explored.

Very few studies to date have looked at the impact of scan length on the quantification of ^90^Y in PET imaging. One such study by Goedicke et al. [22] predicted that an acquisition time of 15 min per bed position should be adequate in order to perform ^90^Y dosimetry; however, they did not explore the extent to which scan time impacted upon image quantification or image quality.

Quantitative imaging requires the maximum possible amount of information to be recovered and retained from the object within the acquisition and reconstruction process. This type of imaging is not necessarily the most visually pleasing, but it does allow more accurate information to be extracted for quantitative calculations.

The purpose of this work was to identify the optimal beta value to use for quantitative ^90^Y PET imaging and to explore the effect that scan length has on quantification; it was hypothesised that increasing the scan length could potentially improve the quantification of PET imaging.

## Materials and methods

### Phantom acquisitions

The NEMA phantom was used to evaluate the effect of both scan duration and the weighting of the penalisation term in the reconstruction on quantification. The phantom was filled following the protocol laid out by the international QUEST study, a multicentre study set up to investigate and compare quantitative ^90^Y imaging on a variety of PET/CT imaging systems [21]. It was filled with an 8-to-1 sphere-to-background ratio of 3160 MBq ^90^Y solution; all 6 spheres with varying diameter (10, 13, 17, 22, 28, and 37 mm) were filled with activity.

The phantom was initially scanned at a high activity of 3020 MBq then left to decay and scanned again at a lower activity of 1040 MBq, which is more representative of the average administered activity for patients undergoing SIRT treatment in Oxford. For both these acquisitions, the phantom was scanned for 10 h overnight on a GE Discovery 710 PET/CT camera (GE Healthcare, Milwaukee), with time-of-flight capability. A single bed position was used for the acquisitions with the spheres in the centre of the image to allow the data to be rebinned into multiple time frames and multiple repeats. The CT was obtained using a pitch of 0.984, 120 kV, automA, and a noise index of 25.

### Reconstruction optimisation

Both overnight NEMA phantom acquisitions were initially rebinned into 15 min frames to match the current clinical acquisition protocol and allow statistical analysis to be carried out. The first 10 concurrent frames were then reconstructed using BPL reconstruction (Q.Clear) with the following range of beta values: 1, 400, 800, 1000, 1200, 1400, 1600, 1800, 2000, 3000, 4000, and 8000. All image analysis was carried out using Hermes Hybrid Viewer (version 2.5, Hermes Medical Solutions AB, Stockholm). Recovered activity percentage (RAP) was calculated for all images; this was achieved by delineating volumes of interest (VOI) around each sphere using the CT image; the size of each VOI was specifically chosen so that it matched the inner diameter of each the sphere in the phantom. These VOIs were then copied across to the co-registered PET images, and the mean pixel value in each VOI was divided by the known sphere activity concentration to give the RAP for each sphere. Contrast recovery (CR) and background variability (BV) were both calculated using these same VOIs for all images, in adherence with the NEMA standard. Further analysis was carried out for acquisition lengths of 30 and 60 min, on a subset of beta values (1000, 2000, 4000), to assess if the optimum beta value was independent of scan time.

### Timing optimisation

Current local clinical protocol for scanning patients post-SIRT is to use 2-bed positions each acquired over a period of 15 min with a 23% overlap. The overnight NEMA phantom acquisitions were rebinned into varying lengths of time (5, 10, 15, 20, 25, 30, and 60 min); 10 repeats of each were acquired in order for uncertainties and statistical analysis to be carried out. All of these rebins were then reconstructed using the beta value that had been identified as optimum for quantification. The same image analysis methodology was used as for the reconstruction optimisation.

### Statistical analysis

All uncertainties in results were calculated using the standard deviation of the mean. The statistical significances between results were determined using two-tailed paired *t* tests with a confidence interval set to 95% and a significance level of 0.05.

## Results

### Reconstruction optimisation

The effect of changing the beta value in the BPL reconstruction was investigated to assess how this would impact quantification and image noise. The 10-mm and 13-mm spheres were excluded from results as the uncertainties in measurements were too high for reliable comparative analysis between different beta values.

**Fig. 1:**
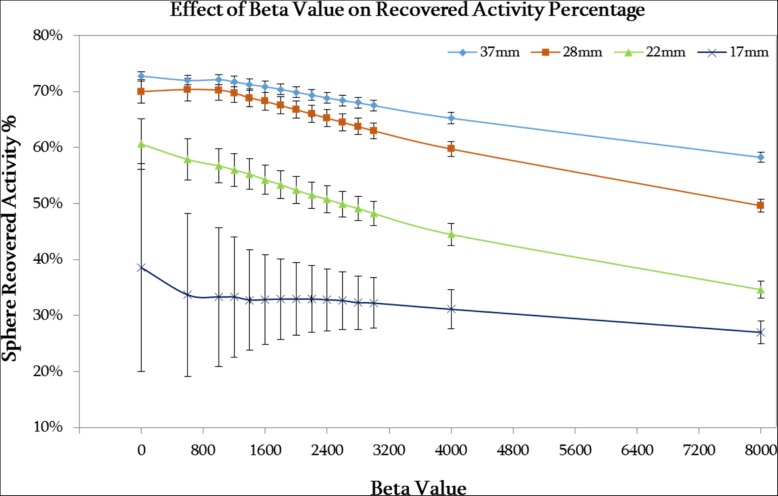
A plot of recovered activity percentage (RAP) against beta value for the 37-, 28-, 22-, and 17-mm-diameter spheres in the NEMA phantom for the low activity (1 GBq) acquisition. Error bars shown are the standard error of the mean

Figure 1 demonstrates that an increase in beta value leads to a decrease in RAP; this results from a higher noise suppression weighting (beta) within the reconstruction. The RAP values for the larger spheres (37 and 28 mm) are much higher than the smaller spheres (22 and 17 mm) owing to larger objects being less affected by the partial volume effect. At lower beta values, the uncertainty in measurement increases, particularly for the 17-mm sphere. Similar results are demonstrated in Additional file 1: Figure S1 for the higher activity (3 GBq) phantom acquisition.

**Fig. 2:**
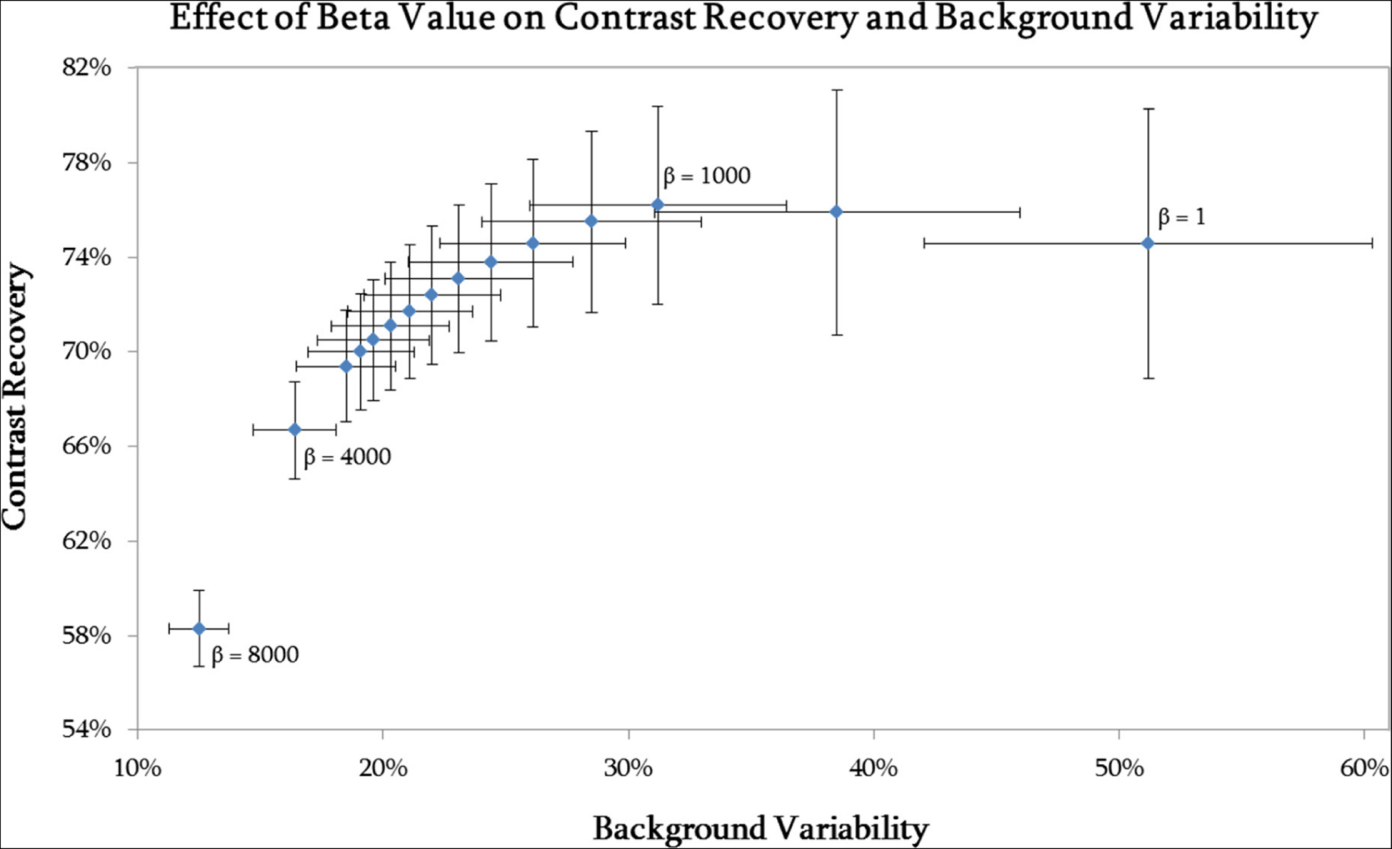
Background variability plotted against contrast recovery for varying beta value (left to right beta = 8000, 4000, 3000, 2800, 2600, 2400, 2200, 2000, 1800, 1600, 1400, 1200, 1000, 600, 1), for the 37-mm-diameter sphere in the NEMA phantom for the low activity (1 GBq) acquisition. Error bars shown are the standard error of the mean

Figure 2 demonstrates that a negative consequence of using a lower beta value is the increase in image noise that occurs. As the beta value decreases, the CR increases and then flattens off; for beta values lower than 1000, there is no increase in CR but a substantial increase in BV. The optimum beta to use for quantification is one that would give the highest CR and RAP without overly accentuating the image noise.

*P* values for differences in RAP are shown in Table 1 for beta values of 1, 1000, 2000, 3000, and 4000 and for the four sphere diameters (37, 28, 22, and 17 mm) on which analysis had been performed. The significance of each result is shown.

**Table 1:** *p* values from the two-tailed paired *t* testing on RAP values for reconstructions with varying beta. Results are shown for the low activity (1 GBq) NEMA phantom acquisition for all analysed spheres

Beta value	Sphere size (mm)	1	1000	2000	3000
1000	37	0.618			
	28	0.929			
	22	0.493			
	17	0.817			
2000	37	0.039*	0.111		
	28	0.233	0.031*		
	22	0.123	0.238		
	17	0.779	0.979		
3000	37	0.001^†^	0.003^†^	0.107	
	28	0.014*	0.005^†^	0.091	
	22	0.023*	0.029*	0.223	
	17	0.744	0.934	0.928	
4000	37	0.000^†^	0.000^†^	0.004^†^	0.125
	28	0.001^†^	0.000^†^	0.003^†^	0.114
	22	0.004^†^	0.002^†^	0.020*	0.208
	17	0.698	0.866	0.871	0.850

*p < 0.05

^†^*p* < 0.01

**Fig. 3:**
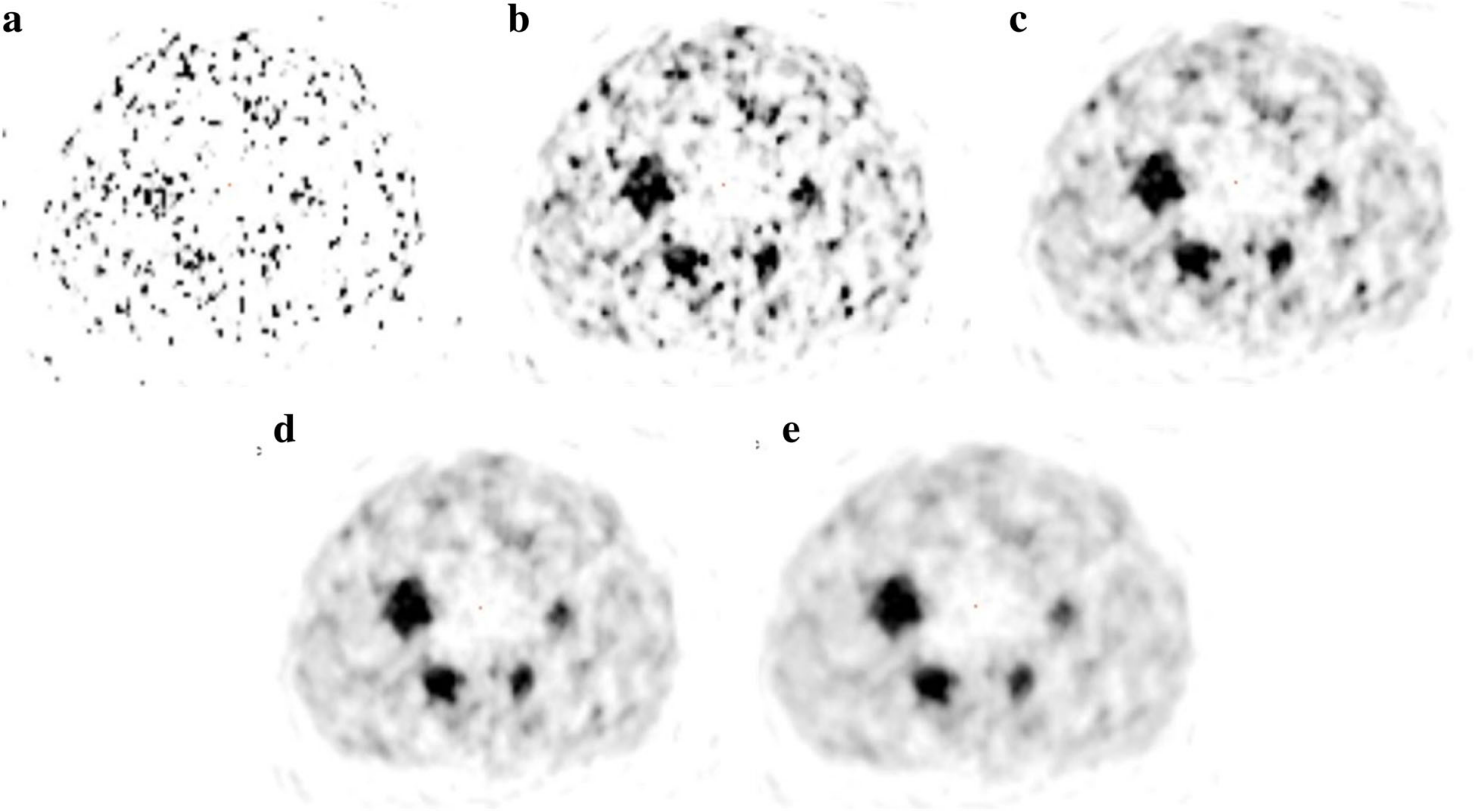
Transaxial views through the centre of the spheres in the NEMA phantom when reconstructed using a variety of beta values. **a** Beta = 1. **b** Beta = 1000. **c** Beta = 2000. **d** Beta = 3000. **e** Beta = 4000

There is no statistically significant difference (*p* > 0.05) in RAP between a beta value of 1000 and 1; it can therefore be stated that there is no improvement in quantification when using a beta value of less than 1000. The benefit of using a beta value of 1000 over a lower value is the reduction in image noise without compromising quantification; this is demonstrated in Fig. 2. For beta values of over 1000, there is a significant reduction in RAP (*p* < 0.05) for at least one out of the three spheres; hence, using a beta value of greater than 1000 would reduce the quantitative accuracy in the image when used in the reconstruction. It is for these reasons that a beta value of 1000 was chosen as the optimum to use in terms of image quantification. NEMA phantom images, reconstructed with varying beta values, are shown in Fig. 3. This visually demonstrates how a higher weighted noise suppression term within the reconstruction effects the resulting image.

**Fig. 4:**
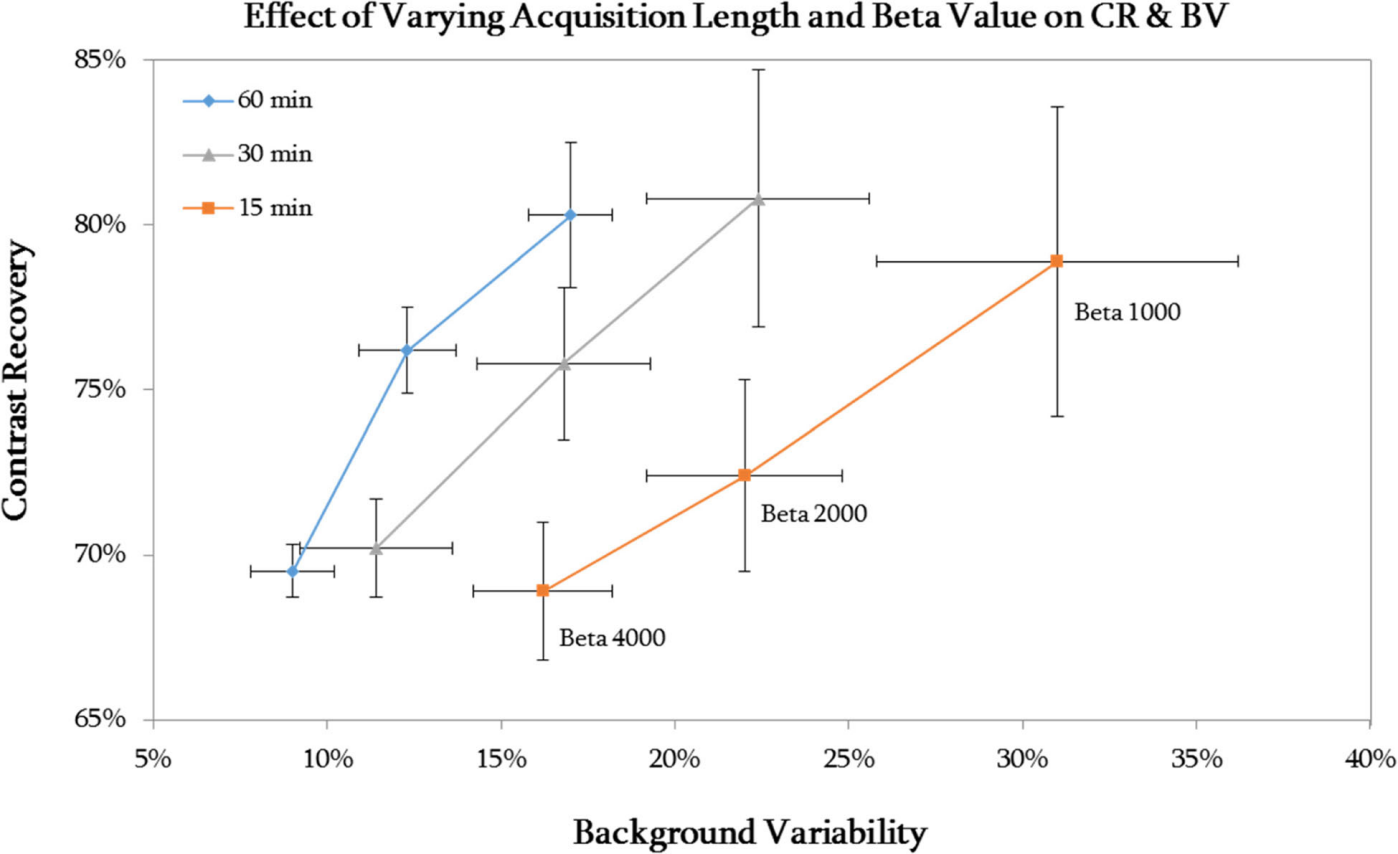
BV plotted against CR for varying beta value and acquisition length. Data is shown for the 37-mm sphere in the NEMA phantom for the low activity (1 GBq) acquisition. Error bars represent the standard error of the mean

Figure 4 shows the relationship between CR and BV for a subset of beta values over a range of acquisition lengths. The same relationship between beta value and CR and BV can be seen for all acquisition lengths, demonstrating that the optimum beta value of 1000 is independent of scan time.

### Timing optimisation

Following on from the optimisation of BPL reconstruction for quantification, an investigation was carried out in order to see if changing the scan time had any impact on quantification. The rebinned overnight acquisitions of the NEMA phantom were analysed for this purpose; the optimum beta for quantification that had been identified (1000) was used for reconstructing each rebin.

**Fig. 5:**
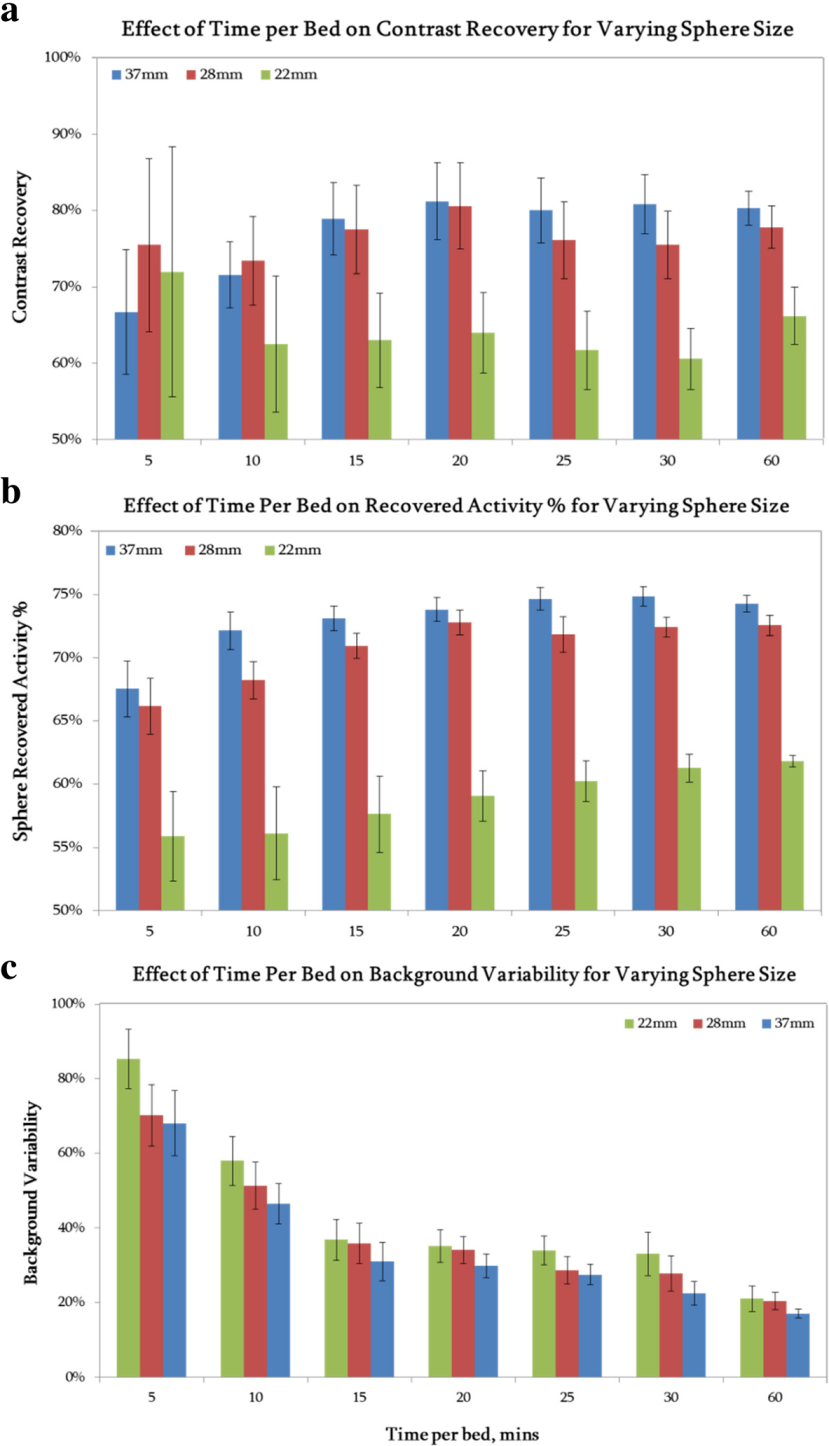
Graphs of **a** CR, **b** RAP, and **c** BV plotted against acquisition length for the low activity (1 GBq) overnight NEMA phantom scan; all images were reconstructed using a beta value of 1000, and results for the three largest spheres are shown. Error bars represent the standard error of the mean

Figure 5 shows the impact of scan time on CR, RAP, and BV for the 37-, 28-, and 22-mm spheres in the NEMA phantom. All error bars displayed are the standard error of the mean measurement. The large uncertainties in CR values at shorter acquisition times are mainly caused by the high level of noise in the background region. Measuring RAP is a more direct assessment of absolute quantification, and it is less affected by image noise due to it being independent of background measurement. Additional file 2: Figure S2 shows these same graphs, but for the high activity (3 GBq) phantom acquisition, similar relationships between CR, RAP, and rated.

*P* values for differences in RAP across varying acquisition lengths and sphere sizes are shown in Table 2. The high uncertainty in results from the 22-mm-diameter sphere meant there was no significant difference (*p* > 0.05) in RAP between any of the time-varying acquisitions for this sphere size. There was also no statistically significant increase in the RAP for any of the spheres for scan times of more than 15 min (*p* > 0.05). The 15-min acquisition produced a significantly higher RAP (*p* < 0.05), for the 37- and 28-mm spheres, than the 5-min acquisition. Whereas for the 10-min acquisition, there was no significant improvement (*p* > 0.05) in the RAP measured for either sphere size when tested against the 5-min acquisition. The 15-min acquisition produces a significantly lower BV (*p* < 0.05) compared to both the 5- and 10-min acquisitions for all sphere sizes. Beyond 15 min, increasing the scan length produces no significant improvement in BV (*p* > 0.05). Additional file 3: Table S1 shows the same statistical analysis carried out for the high activity (3 GBq) phantom acquisition. Similarly, above 15 min, there is no statistically significant improvement in RAP.

**Table 2:** *p* values from the two-tailed paired *t* testing on RAP values for acquisitions with varying scan time. Results are shown for the low activity (1 GBq) NEMA phantom acquisition for all analysed sphere sizes

Time per bed (mins)	Sphere size (mm)	5	10	15	20
10	37	0.102			
	28	0.449			
	22	0.642			
15	37	0.034*	0.592		
	28	0.047*	0.134		
	22	0.523	0.698		
20	37	0.019*	0.356	0.608	
	28	0.013*	0.019*	0.192	
	22	0.486	0.561	0.389	
30	37	0.006^†^	0.119	0.169	0.405
	28	0.013*	0.021*	0.246	0.769
	22	0.164	0.198	0.235	0.256

*p < 0.05

^†^*p* < 0.01

Fifteen minutes is the shortest acquisition length where an increase in scan time does not produce a statistically significantly higher RAP.

**Fig. 6:**
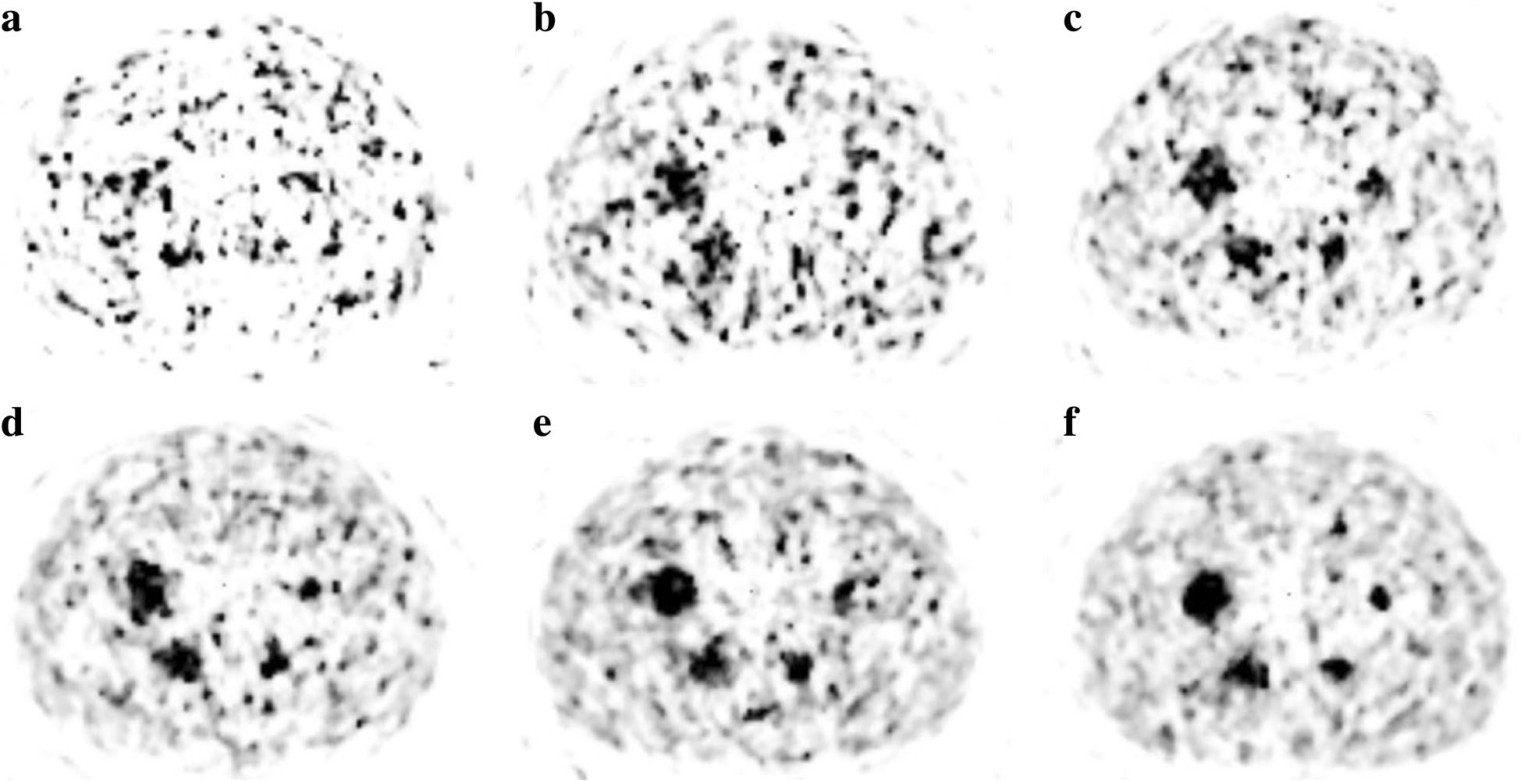
Transaxial views through the centre of the spheres in the NEMA phantom for variety of acquisition lengths. **a** 5 mins. **b** 10 mins. **c** 15 mins. **d** 20 mins. **e** 25 mins. **f** 30 mins. A beta value of 1000 was used for all reconstructions

**Fig. 7:**
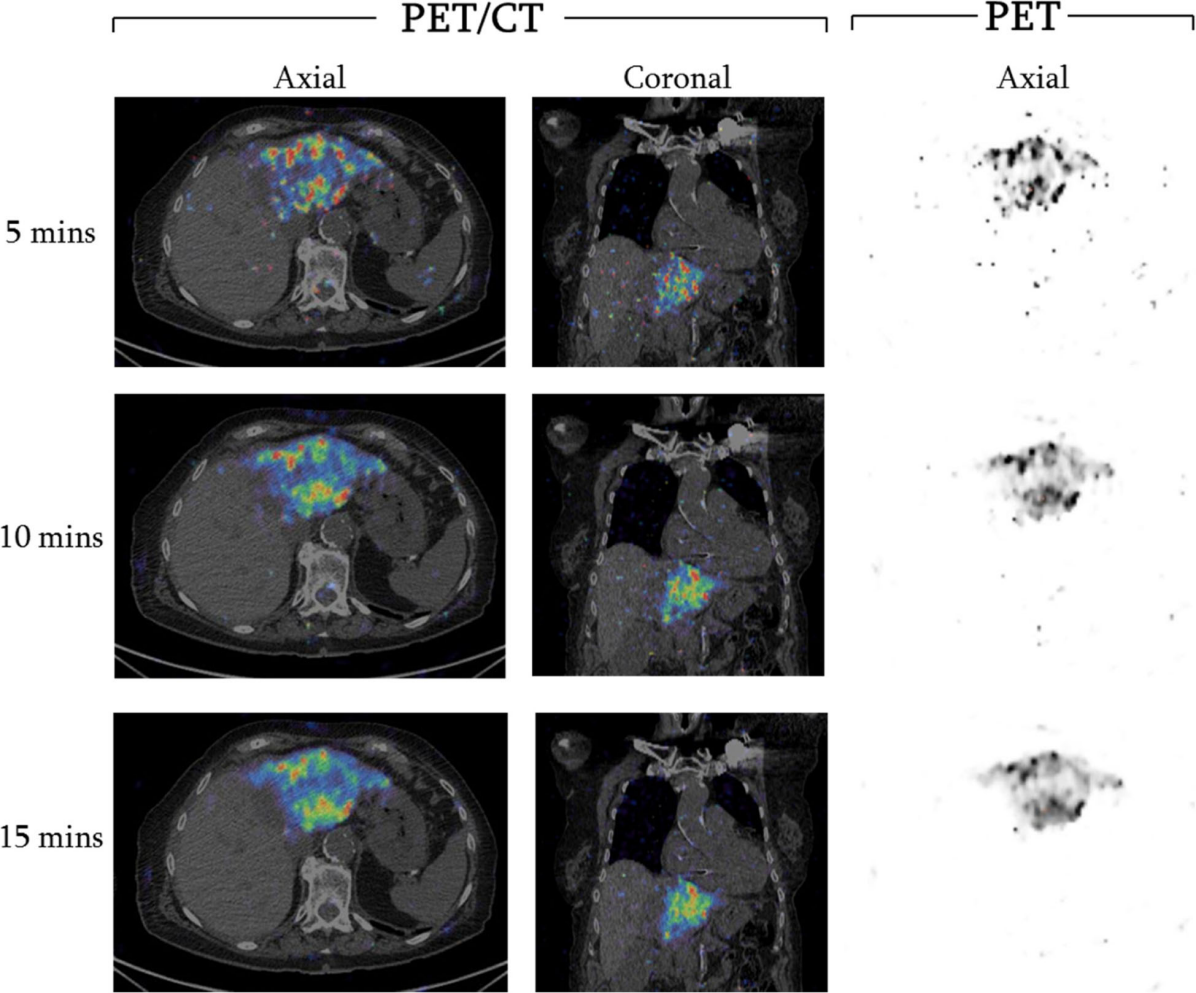
Images of varying acquisition lengths for a patient who had a PET/CT scan post-SIRT. Reconstructions have been performed using BPL with a beta value of 1000. Axial and coronal slices of the fused PET/CT images are displayed as well as axial slices of just the PET images. The same slices have been used for all acquisition lengths

A visual demonstration of how the acquisition length affects the phantom image is shown in Fig. 6; for the two shortest acquisitions, it is more difficult to visualise the spheres and the background is exceptionally noisy. A post-SIRT PET/CT scan of a patient has also been retrospectively reconstructed (using a beta value of 1000) for varying acquisition lengths; this is displayed in Fig. 7. It can be seen that a greater amount of activity is recovered as the acquisition length increases, and the noise in the images, particularly outside the liver, decreases.

## Discussion

The aim of this investigation was to optimise both the reconstruction and acquisition length of imaging ^90^Y in PET/CT, with the purpose of providing an optimised protocol for quantification. This was done with a view to using PET/CT for post-SIRT dosimetry, in line with the new EU directive [23].

The optimum weighting of the noise penalisation term in the BPL reconstruction algorithm was determined through the use of phantom-based measurements, at both an activity representative of the average administered activity for SIRT patients, and a higher activity acquisition representing the upper end of administered activities. It was demonstrated that a beta value of 1000 provided the best image quantification; it yielded the highest CR of 75%, and there was no significant improvement in RAP for lower beta values. This validates the work carried out by Rowley et al. who hypothesised that a lower beta value would produce more quantitatively accurate images than using a beta value of 4000—which is optimum for visualisation [21]. Higher beta values provide a lower image noise which is expected due to the nature of the noise suppression term in the reconstruction algorithm. A low image noise is not particularly important when imaging purely for the purpose of quantification but is more ideal for visualisation. A beta value of 1000 allowed maximum quantification to be achieved without overly accentuating the noise; this was also shown to be true across a range of acquisition lengths. For beta values < 1000, there was no improvement in quantification but a large increase in noise.

The NEMA phantom images for varying beta values, shown in Fig. 3, visually demonstrate the decreasing image noise as the beta value increases. Although the images with higher beta values (> 1000) look more aesthetic and clear due to the lower amount of noise, they are not as quantitatively accurate as has been demonstrated in this work.

The optimum acquisition length of ^90^Y PET imaging was also determined using phantom measurements. Current local protocol scans patients for 15 min per bed position; it was thought that this length of time could be increased to improve quantification or even reduced if it did not impact on quantification. From both a logistical and patient perspective, scanning for the minimum possible time is ideal; this limits movement artefacts as well as patient discomfort and maximises patient throughput. Interestingly, results showed that increasing the length of time per bed had no statistically significant impact on quantification; there was no significant increase in CR or RAP. However, reducing the acquisition time to less than 15 min per bed decreases the quantification accuracy of the image and significantly increases the noise in the image. As no improvement in quantification, and only a reduction in noise, was observed for acquisition times greater than 15 min, and quantification decreased for shorter acquisition times, 15 min was chosen as the optimum time per bed to use for post-SIRT PET imaging.

SIRT patients are administered with a range of different activities, so it was important to show that results were valid for both higher and lower activity acquisitions, here 3 GBq and 1 GBq, respectively. By scanning the phantom at two different activities, any dependence of beta value or scan length on activity could be assessed. The results demonstrate that a beta value of 1000 is optimum for quantification in both cases and that a 15-min acquisition is the minimum scan time necessary to achieve sufficient quantification, again in both cases.

Based on these results, a recommendation of scanning patients for 15 min per bed and performing BPL reconstructions using a beta value of 1000 is made for quantitative ^90^Y imaging.

## Conclusion

PET/CT has been optimised for quantitative post-SIRT imaging of ^90^Y, both in terms of acquisition length and reconstruction. It was found that using a beta value of 1000 within Q.Clear reconstruction provided the best quantification, using a value lower than this only increased the image noise. An acquisition length of 15 min per bed was found to be optimum for quantification; increasing the acquisition time reduced the image noise but did not provide any statistically significant improvement in quantification.

## Additional files

Additional file 1: **Figure S1.** A plot of recovered activity percentage (RAP) against beta value for the 37-, 28-, 22-, and 17-mm-diameter spheres in the NEMA phantom for the high activity (3 GBq) acquisition. Error bars shown are the standard error of the mean. (DOCX 69 kb)

Additional file 2: **Figure S2.** Graphs of a) CR, b) RAP, and c) BV plotted against acquisition length for the high activity (3 GBq) overnight NEMA phantom scan; all images were reconstructed using a beta value of 1000 and results for the three largest spheres are shown. Error bars represent the standard error of the mean. (DOCX 293 kb)

Additional file 3: **Table S1.** *p* values from two-tailed paired *t* testing on RAP values for acquisitions with varying scan time. Results are shown for the high activity (3 GBq) NEMA phantom acquisition for all analysed sphere sizes. (DOCX 13 kb)

## Abbreviations

BPL: Bayesian penalised likelihood
BSREM: Block sequential regularised expectation maximisation
BV: Background variability
CR: Contrast recovery
CT: Computed tomography
EU: European Union
FDG: Fluorodeoxyglucose
MAA: Microaggregated albumin
NEMA: National Electrical Manufacturers Association
OSEM: Ordered subset expectation maximisation
PET: Positron emission tomography
RAP: Recovered activity percentage
SIRT: Selective internal radiation therapy
VOI: Volume of interest
