# Supplementary figures and images for: Optimising quantitative 90Y PET imaging: an investigation into the effects of scan length and Bayesian penalised likelihood reconstruction
Source: EJNMMI Res. 2019 May 10;9:40. doi: 10.1186/s13550-019-0512-y (PMC6510762; doi:10.1186/s13550-019-0512-y)

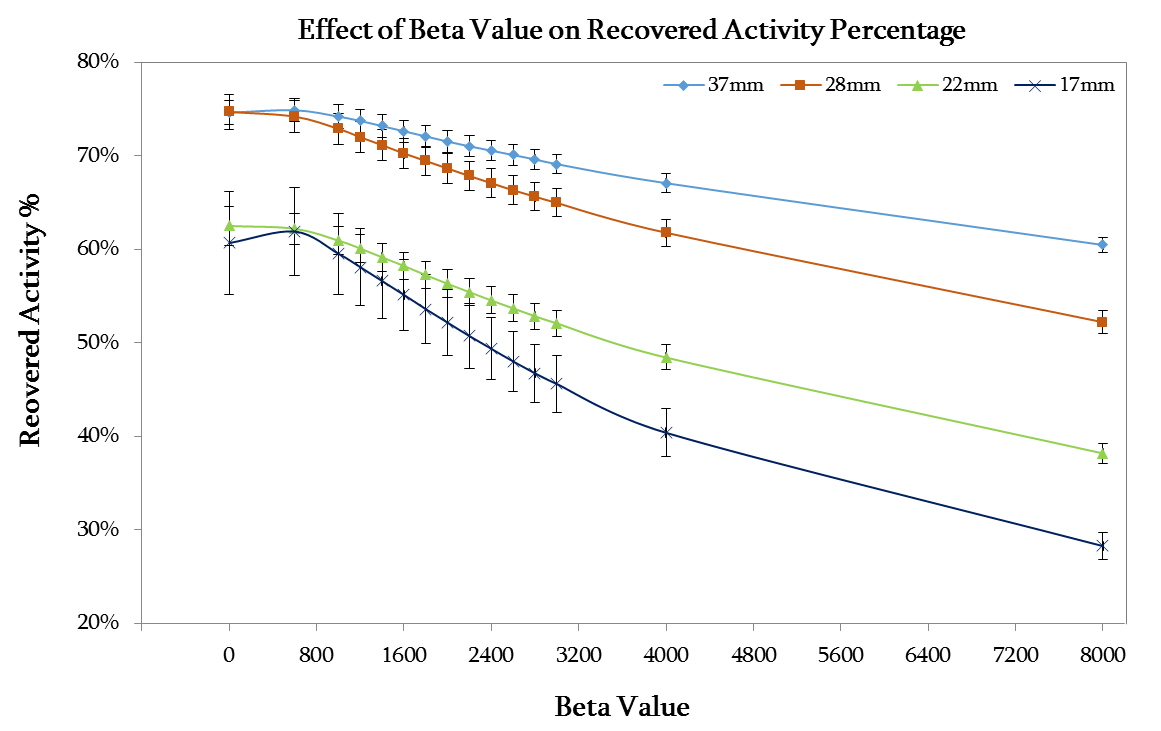

Supplement: Supplementary file 1 — Figure S1. A plot of recovered activity percentage (RAP) against beta value for the 37-, 28-, 22-, and 17-mm-diameter spheres in the NEMA phantom for the high activity (3 GBq) acquisition. Error bars shown are the standard error of the mean. (DOCX 69 kb) [file 13550_2019_512_MOESM1_ESM.docx]

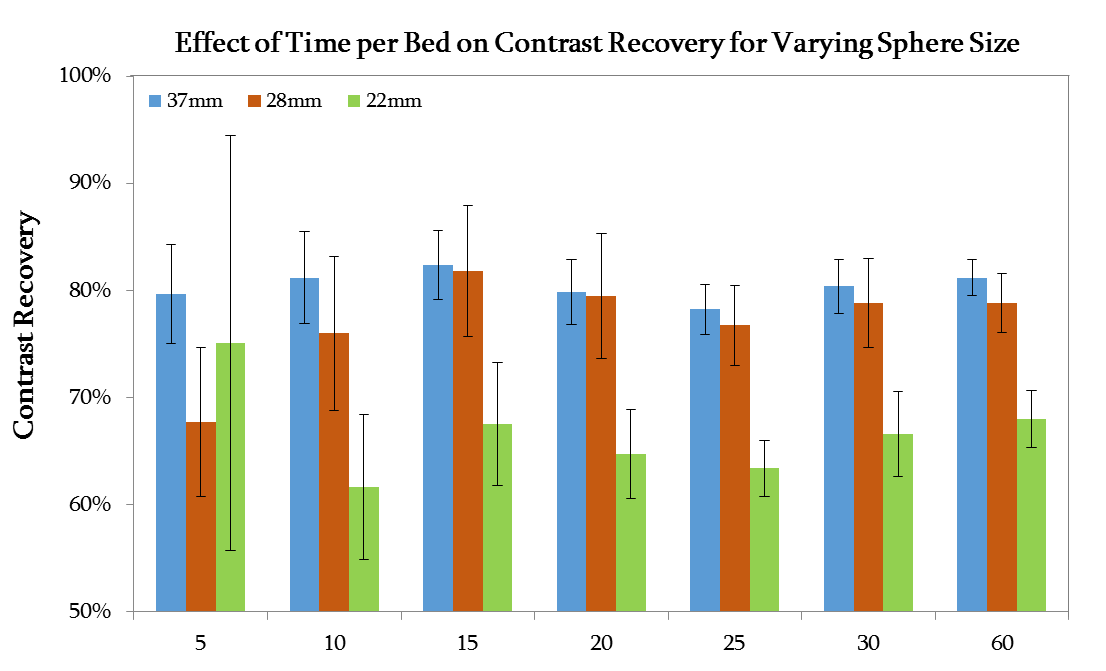

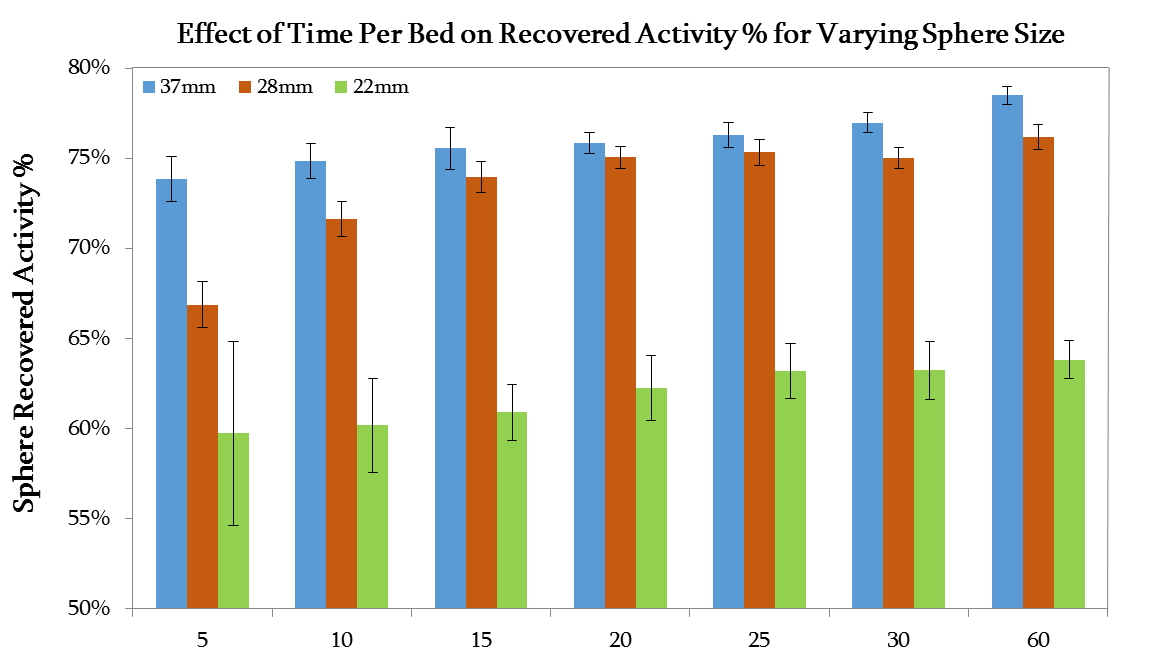

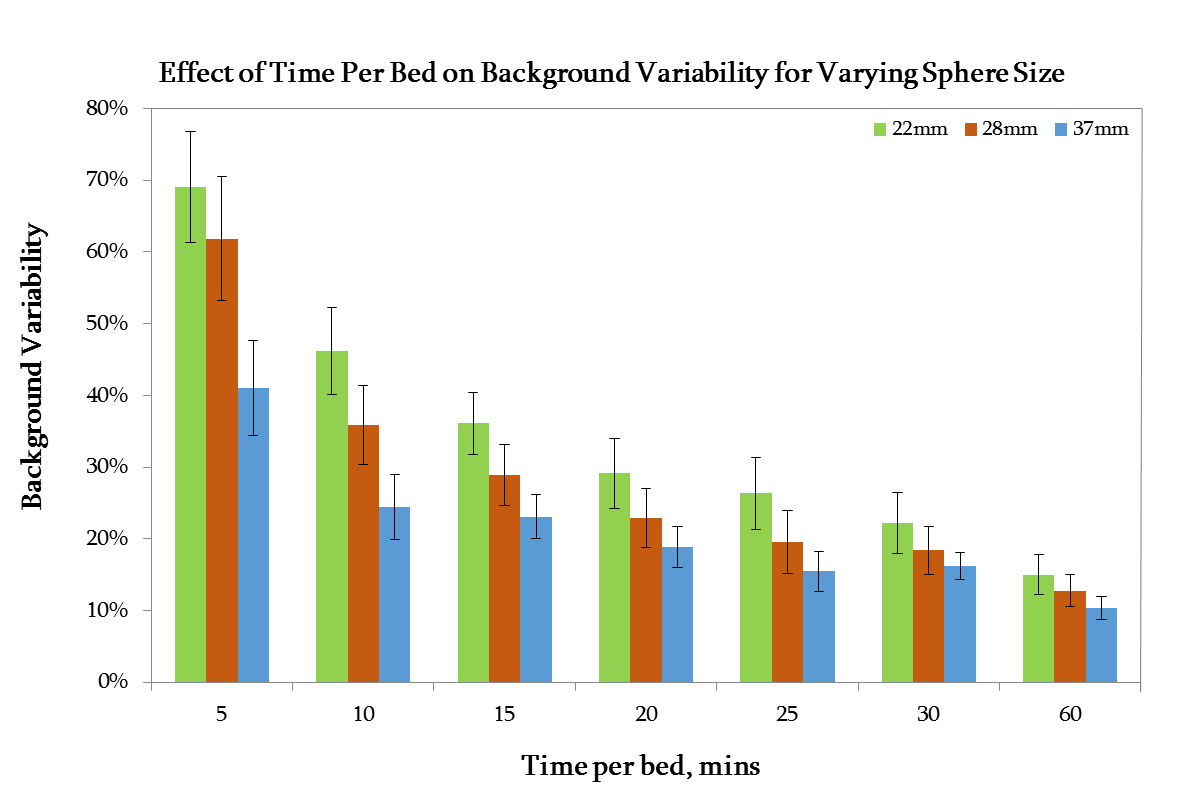


**a)**

**b)**

**c)**

Supplement: Supplementary file 2 — Figure S2. Graphs of a) CR, b) RAP, and c) BV plotted against acquisition length for the high activity (3 GBq) overnight NEMA phantom scan; all images were reconstructed using a beta value of 1000 and results for the three largest spheres are shown. Error bars represent the standard error of the mean. (DOCX 293 kb) [file 13550_2019_512_MOESM2_ESM.docx]
