# Supplementary material for: Optimising quantitative 90Y PET imaging: an investigation into the effects of scan length and Bayesian penalised likelihood reconstruction
Source: EJNMMI Res. 2019 May 10;9:40. doi: 10.1186/s13550-019-0512-y (PMC6510762; doi:10.1186/s13550-019-0512-y)
Supplement: Supplementary file 3 — Table S1. p values from two-tailed paired t testing on RAP values for acquisitions with varying scan time. Results are shown for the high activity (3 GBq) NEMA phantom acquisition for all analysed sphere sizes. (DOCX 13 kb) [file 13550_2019_512_MOESM3_ESM.docx]

**Supplementary Table 1** P-values from 2-tailed paired t-testing on RAP values for acquisitions with varying scan time. Results are shown for the high activity (3 GBq) NEMA phantom acquisition for all analysed sphere sizes.

| **Time-per-bed (mins)** | *Sphere Size, mm* | **5** | **10** | **15** | **20** |
| --- | --- | --- | --- | --- | --- |
| **10** | *37* | 0.527 |  |  |  |
|  | *28* | 0.008*^†^* |  |  |  |
|  | *22* | 0.940 |  |  |  |
| **15** | *37* | 0.336 | 0.662 |  |  |
|  | *28* | 0.000*^†^* | 0.086 |  |  |
|  | *22* | 0.831 | 0.815 |  |  |
| **20** | *37* | 0.168 | 0.402 | 0.820 |  |
|  | *28* | 0.000*^†^* | 0.007*^†^* | 0.340 |  |
|  | *22* | 0.648 | 0.519 | 0.577 |  |
| **30** | *37* | 0.037*^*^* | 0.080 | 0.292 | 0.202 |
|  | *28* | 0.000*^†^* | 0.007*^†^* | 0.343 | 0.980 |
|  | *22* | 0.506 | 0.506 | 0.310 | 0.689 |
| ** p<0.05, ^†^p<0.01* | |  |  |  |  |
